# Sustainability of Four Dairy Farming Scenarios in an Alpine Environment: The Case Study of Toma di Lanzo Cheese

**DOI:** 10.3389/fvets.2020.569167

**Published:** 2020-10-09

**Authors:** Tibor Verduna, Simone Blanc, Valentina Maria Merlino, Paolo Cornale, Luca Maria Battaglini

**Affiliations:** Department of Agricultural, Forest and Food Sciences, University of Turin, Turin, Italy

**Keywords:** cheese production, dairy farming, human-edible feed conversion efficiency, life cycle assessment, life cycle costing, mountain environment, sustainability

## Abstract

The dairy sector accounts for a large share of all European agricultural production, at the same time however, it is one of the most ascribed sector contributors to the environmental impact of agriculture, particularly for greenhouse gas emissions. Simultaneously, it is a strategic sector for the economy but generates increasing debate in the community regarding the social aspects mainly related to the use of resources and the food-feed competition of livestock involving the reduction of human-edible crops. In this general framework, this study aims to compare four different dairy farming scenarios characterized by different use of environmental resources in the Alpine area, considering as a case study the production of the Toma di Lanzo cheese (a traditional cheese produced in the mountainous regions of Piedmont—Northwest Italy). The study envisaged the integrated use of three methodologies: Life Cycle Assessment, Life Cycle Costing and the assessment of human-edible feed conversion efficiency to jointly analyze environmental, economic and social aspects. The main results of this research highlighted how the utilization of local breeds, which maximize the efficiency in the use of territory resources, such as grasslands in a mountain environment, allowed dairy production to reduce emissions, when compared to the high-input traditional breeding systems. Although the mountain livestock systems have several critical issues mainly linked to social factors such as low generational turnover, work schedules, modest life quality of families, it is however possible to earn more income than in lowland scenarios. At the same time, this production system allows the Toma di Lanzo cheese-making heritage to be preserved. This mountain pasture cheese, to which superior organoleptic and nutritional characteristics are attributable, when compared to cheeses from the valley floor, incorporates traditional values, a link to the territory and the transmission of knowledge. With reference to food-feed competition in livestock involving the reduction of the use of human-edible crops and feedstuffs in animal diets, we found that grazing and grass-based feeding systems were one of the most sustainable ways to produce milk.

## Introduction

The livestock sector, one of the most important parts of the agrarian economy at European level, is charged as being the major contributor to the environmental impact of agriculture, more specifically due to greenhouse gas (GHG) emissions (1–3). The livestock sector is generally considered responsible for a significant negative impact on the environment due to the considerable production of wastewater and effluents with a high pollution rate (2, 4). Overall, although emissions from the agricultural sector have decreased over the last 20 years in EU countries, the dairy production chain alone has considerably increased methane (CH_4_) emissions by 22.5%, between 1990 and 2017 (5). Over the last decade, this negative impact on sustainability has been directly linked to the increase in herd density and the need to improve the performance of livestock production, to meet the growing animal-based food demand for human consumption (6–9).

In Italy, 70% of CH_4_ emissions from the agricultural sector derive from the enteric fermentation by animals in intensive farming and 20% from the management of manure and slurries (10). In this scenario, Italy is in third place, after France and Germany, respectively, for the contribution of enteric fermentation deriving from the cow milk production sector. These data are probably related to the characteristics of the modern Italian high-input intensive systems of the milk supply chain. In fact, over the years, a structural change has been underway, leading to a reduction in farm numbers tied to a steady upward trend in animal density. In Italy, at the end of 2019, dairy cows totaled 2,612,729 heads (11) for an annual milk production delivered to dairies of 12,112,000 tons, equal to ~8% of the total European production (158,257,000 tons in 2019) (12).

These numbers have led to a greater awareness and need to reduce global greenhouse gas emissions in order to curb climate change, through the mitigation of environmental impacts and focusing on a greater economic and environmental sustainability of human activities (5). However, this sector, characterized by an extreme intensification of livestock farming, has a considerable economic impact, with a supply chain worth 58 billion euros in 2018 (14% of European agricultural production) (13). The need to increase the sustainability of these productions could possibly be achieved by optimizing the management of the farming system, through the exploitation of available marginal areas (14). This development is already rooted in some areas of Italy, where dairy farming in marginal areas is not only a tradition, but also an instrument for the enhancement of territories, farms and products (15). Of the total number of dairy cows reared in Italy, 22,085 are raised in 288 farms following the practice of transhumance (11). Of these, 268 farms (21,130 heads) are located in Piedmont (Northwest Italy), where extensive dairy cattle rearing and transhumance has been rediscovered by exploiting the link and proximity to the Alpine environment. Moreover, the intensification of Alpine agroecosystems and associated ecosystem services provides socio-economic positive impacts, in addition to the development of sustainable production methods (16, 17). The vertical transhumance of ruminants (cows, in addition to goats, and sheep) in summer, from permanent farms in the valleys to temporary farms in Alpine pastures, represents one of the most distinctive and important traditional activities for both landscape preservation and the production of typical local products.

The Italian dairying tradition produces a wide variety of cheeses strongly related to their place of origin. In addition to well-known protected designation of origin (PDO) cheeses, there are also numerous historical and traditional cheeses. These products fall into the Italian category of Traditional Agricultural Food Products (PAT, *Prodotto Agroalimentare Tradizionale*), and their entire production process follows traditional rules. They are characterized by being produced from a small number of manufacturers in a confined region, with a highly variable production and a limited number of final rounds.

The assessment of the environmental impact of mountain production systems must not only take into account milk production, but also the different ecosystem services connected with it. In fact, these systems are characterized by rearing of local hardy breeds, not suitable for high-input intensive systems, and have the ability to exploit and optimize the fodder resources and pastures, unique to these areas. In this regard, animal production in these areas is also characterized by a reduced (or in some cases zero) use of human edible resources. This reduced intake is significant in light of the growing concern regarding human food supply.

In literature, several researches have focused on the assessment of the environmental and economic impacts of the Italian dairy chain by using the Life Cycle Thinking (LCT) approach. Within the LCT approach, many different methodologies are useful to evaluate the environmental and economic impacts of a life cycle, and the commonly used methodologies are Life Cycle Assessment (LCA) and Life Cycle Costing (LCC) (18–21).

The LCA tool allows the evaluation of the environmental impact generated during the life cycle of a product or a service following the Principles and Frameworks of UNI EN ISO 14040:2006. According to this ISO standard, throughout the product lifecycle, LCA allows the input and output flows of energy and materials to be recorded and evaluated, in addition to the potential environmental impacts of a product or a service. This standardized procedure identifies the environmental consequences of a product life cycle, by assessing the impacts generated by the entire production chain (22), from inputs to the final product, as well as simultaneously assessing different impact categories including greenhouse gas emissions, acidification, eutrophication, land use and energy consumption (23). LCA has also been used in previous researches as a tool to compare the efficiency of alternative management systems implemented along the livestock supply chains, in order to reduce the environmental impact, when compared to traditional-intensive production systems (24).

LCC allows the costs related to each phase of the life cycle to be analyzed. This methodology is often used in agro-food sector research, also examining the issue of revenues obtained from the sale of products, so that the profits generated by the different examined scenarios can also be identified. Therefore, similar to the LCA analysis, the impact assessment phase is replaced by the computation of profitability.

Regarding the livestock sector, several research articles published in recent years have evaluated the costs, sometimes in association with environmental aspects. Among these, we report those related to the analysis of the cost of production of forage to feed dairy cattle in Northern Italy (25), and to the definition of costs to regulate ammonia emissions from livestock farms in Germany, the Netherlands and Denmark (26). The paper authored by Cecchini et al. (27) analyzed the environmental efficiency of carbon dioxide abatement costs in dairy cattle farms in Italy, associated with efficiency performance measures. In a similar way another study evaluated the effect of animal nutrition and grazing on GHG emissions with reference to milk production and the related farm income (28).

## Aims

The development of more environmentally sustainable farming systems—based e.g., on the availability of pasture, or the use of alpine pastures—facilitating the reduction of greenhouse gases, also represents an instrument capable of enhancing the production system, the product, in addition to valorizing the producer role. Moreover, in response to increased consumer demand for sustainable products and a return to traditional food products, animal husbandry is focusing on the reinstatement of autochthonous breeds, on territories and on eco-sustainable products (29). In effect, these breeds, territories and products, by way of their characteristics, can be defined as prototypes of sustainability in all its dimensions (environmental, economic, and societal) (30–32). Examining this complex scenario, in conjunction with the social-economic and environmental impact of Alpine dairy production systems, this research aims to develop the following points:

### OBJ 1

Given the importance of products and ecosystem production systems on environmental impact and territorial sustainability, the LCA tool was used to evaluate the environmental impacts, from cradle-to-retail, of the Italian PAT Toma di Lanzo cheese. In literature, several researches have been conducted to evaluate the environmental impact of Italian cheeses using LCA. However, to our knowledge there are no works that deal with a product such as Toma di Lanzo, whose production is strictly dependent on the resources of the territory in which it is produced, so deriving from a model consistent with the Alpine system. The strategies of innovation and enhancement are not connected to the product, but to the unique peculiarities of the production system. In this research, the analysis was performed by comparing the phases of the production process (rearing, milking, cheese making, and transport) in four different scenarios, two high-input-lowland and two low-input-mountain scenarios.

### OBJ 2

According to Genovese et al. (33), there is a certain difficulty in developing system strategies that enhance mountainous territories and their related products (34). A conducive enhancing strategy that can assist in setting forward actions to improve typical productions, is the analysis of economic sustainability, which can be linked to economic analyses. To achieve this goal, LCC was carried out to determine the profitability of the production of Toma di Lanzo, by means of costs and revenues analysis in the four scenarios examined.

### OBJ 3

Minimizing food-feed competition of livestock by reducing the use of human-edible crops and feedstuffs in animal diets represents a promising strategy to increase sustainability in livestock productions (35, 36). From this perspective, a feeding system based only on grass has a different impact than feeding a cereal-based diet, in addition to the amount of supplemented concentrate and the type of feed ingredients (cereal grains vs. by-products) in cow diets. Therefore, the goal is to assess the efficiency of the four examined scenarios in terms of human food production. In the present study, the net contribution of dairy production to the human food supply have been measured using two indicators, the human-edible feed conversion efficiency (37), and the net food production (38).

## Methodology

### Designation of Scenarios

The study took place in the Lanzo valleys, located in Piedmont, Northwest Italy. It involved farms specialized in cow milk and Toma di Lanzo cheese production. The farms were selected to be a representative sample of dairy farms of that region: family run dairy farms, medium size farm, transhumance to mountain pasture in summer, cows diet based on grazing, and conserved forages plus compound feeds. The system boundaries encompassed the phases of grazing, milking, cheese-making and transport in 4 scenarios, determined by the movement of the herd during the year: Indoor Winter Feeding (IWF), Valley Bottom Grazing (VBG), Mountain Pasture Grazing (MPG), and Alpine Pasture Grazing (APG).

The characteristics of the four scenarios are reported in Table 1. The number of observations per scenario is equal to six because all the selected farms are involved in the four scenarios. Two dual-purpose alpine breeds (Aosta Red Pied and Pustertaler-Sprinzen) are mainly reared in the selected farms; only one farm raises crossbreds. The cows of the six herds are kept in tie-stall housing systems and the number of lactating cows is, from the smallest to the biggest farm, 45, 46, 50, 54, 70, and 85, respectively. The six farms involved in the study practice vertical transhumance to mountain pastures during the warmer seasons, whereas during the winter months, cattle are stabled in lowland farms. Their diets consist of meadow hay and compound feeds (i.e., grains, cereal by-products, and occasionally a commercial concentrate). Farmers schedule calving season in the stabled period to avoid the presence of gestating cows and parturitions at mountain pasture. In springtime, cattle are allowed to graze in the meadows surrounding the farm for a few hours a day. Cows are indoor fed with hay and, in some cases, supplemented to sustain milk production. Herds are moved to the mountain pastures in June, where cows are free to graze all day, moving them to different areas according to grass availability. Supplementation, practiced in half of the farms, consists of 1–2 kg of compound feed, distributed in the milking parlor. Then, herds are moved to higher altitude Alpine pastures (~2,000 m a.s.l.) in summer. At the end of the summer season, herds descend to mountain pastures to graze the regrowth in the same areas, before moving back to the lowland farms. The grazing of meadows around the farm is practiced provided that climate conditions are favorable.

**Table 1:** Characteristics of the four scenarios.

	**Indoor winter feeding (IWF)**	**Valley bottom grazing (VBG)**	**Mountain pasture grazing (MPG)**	**Alpine pasture grazing (APG)**
Location	Farm	Lowland pastures	Mountain pastures	Alpine pastures
Altitude (m a.s.l.)	301–875	301–875	1,200–1,800	1,800–2,200
Season	Winter (Dec to Mar)	Spring & Autumn (Apr-May & Oct-Nov)	Summer (Jun & Sep)	Summer (Jul-Aug)
Duration (days)	100–140	90–130	45– 95	30–60
Pasture (ha)	-	35–80	30–110	10–111
Feeding system	Winter feeding (hay + cereals *or* concentrates)	Grazing *or* Grazing+ supplementation	Grazing *or* Grazing+ supplementation	Grazing *or* Grazing+ supplementation
Lactation (wks)	1st−15th & 44th−52nd	16th−23rd & 40th−43rd	24th−27th & 36th−39th	28th−35th

#### Toma di Lanzo Cheese

To better understand the object of this study, a brief description of the physical characteristics and production process of Toma di Lanzo cheese is given.

Toma di Lanzo cheese belongs to the historical cheese making tradition of the Piedmont region (NW Italy) and is now labeled PAT (Traditional Agricultural Food product). Toma di Lanzo is a semi-hard, semi-cooked cheese. The cheeses have a flattened cylindrical shape, 5–15 cm high, 20–40 cm in diameter, and weigh 3–7 kg. The cheese is produced from bovine milk only. Raw milk obtained from one or two consecutive milkings is used. The milk from the evening session is partially skimmed following overnight creaming and then mixed to the whole milk from the next morning milking. Rennet is added to the milk at a temperature of 35–37°C. The milk is then left to rest for about 1 h, but appropriate clotting time is visually established by the cheese-maker. After cutting to the dimension of corn/rice grains, the curd is then heated up to 42°C. Successively, the curd is collected and placed in molds or bound in natural cloths. The cheese is then salted in brine (usually 20% NaCl), but dry salting is also performed. Finally, the cheese is ripened in cellars or natural caves at constant temperature and humidity levels (6–10°C and 85% RH, respectively), lasting 15 days for small cheese rounds or 60 days for larger sizes. The cheese appears ivory-white to yellowish with small and sparse eyes. The texture is soft or slightly compact. The flavor is slightly sweet and milky and becomes stronger and tastier after longer maturation periods.

### Life Cycle Assessment

The environmental impact of Toma di Lanzo cheese was determined by a cradle-to-retail approach, using the LCA method based on the UNI EN ISO 14040:2006. The six dairy farms were analyzed and average data were used in the data processing. This approach involved both milk production at farm level and cheese-making at the dairy plant.

To assess the supply chain, both raw milk and Toma di Lanzo cheese were considered. The functional units used were: 1 kg of FPCM (Fat-Protein Corrected Milk) at farm level and 1 kg of Toma di Lanzo cheese—with a dry matter fat content of 32% and a moisture content of 35–45%—at dairy plant level.

#### System Boundaries and Data Collection

A detailed analysis was conducted, through a field investigation with a questionnaire submitted to the farmers. Each step was characterized by material requirements using data collected at farm and dairy plant level. Concerning the grazing phase, we focused on farm management (herd composition, housing system variation through the scenarios, manure management, ration composition) and data on the input and output mass flows (forage, concentrate, energy, water, milk). For the milking and cheese-making phases, the survey included measurements and information on resources (materials, energy, water) and waste (the whey is also fed to dairy cows). For the transport phase, we collected data about the distance and vehicle.

Production data of milk and cheese were provided both by dairy farms and by the production traceability system of the Toma di Lanzo Consortium.

#### Inventory Analysis

**Figure 1:**
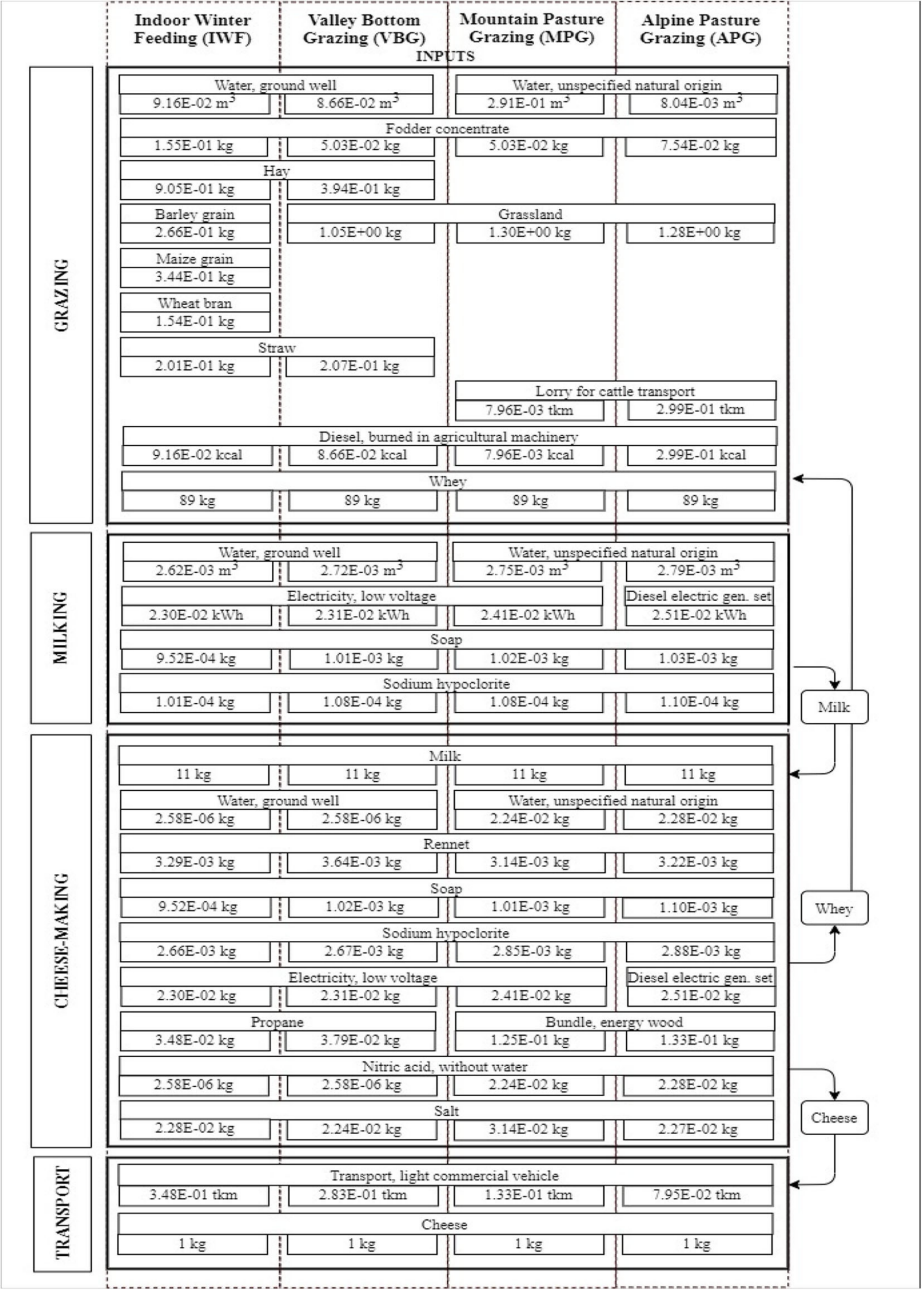
Inventory input from cradle-to-retail and system boundaries. Dotted lines represent the scenarios and black squares the phases.

Figure 1 summarizes the flows and input of grazing, milking, cheese-making and transport of the four scenarios examined. The Life Cycle Analysis included the production of raw milk, farming of fodder, transport and production of raw materials (animal feed, cleaning products, bedding material), consumption of water, energy and fossil fuels, and management of manure, slurry, and wastewater. In the IWF setting, cows were housed in a permanent stall and fed with concentrate, hay, barley grain, maize grain and wheat bran, while in the VBG scenario, they were also left to graze on grass and therefore the amount of concentrate is lower; in MPG and in APG settings, the cows were conducted to mountain pastures and fed with grassland and small amounts of concentrate. In the LCA inventory, only purchased foodstuffs have been included, since the impact of feed production in farms had already been taken into account (land occupation and diesel consumption for farming), in accordance with Laca et al. (39).

Some inputs are common to all scenarios, whereas other inputs are specific. More precisely, in the APG and MPG scenarios, the water used in the grazing phase is spring water, while in the VBG and IWF scenarios it is supplied by a well.

The transport of animals to the Alpine pasture only refers to the APG and MPG scenarios. The quantity and type of externally sourced feedstuffs is different in the four scenarios examined.

The electricity used for milking and cheese-making, is provided by the electric grid for the MPG, VBG and IWF scenarios, while a diesel-electric generating set with diesel consumption was used in the APG scenario, where the dairy plant was not connected to the electric grid.

In the cheese making phase, heat is produced by using electricity in the VBG and IWF scenarios, while a wood-fired boiler is used in the APG and MPG scenarios.

#### Calculation of Emissions to Air, Soil, and Water

Calculation of CH_4_ from enteric fermentation was based both on dry matter intake and the live weight of cows, using the following equation developed by Yan et al. (40):

CH_4_ (L day^−1^) = 0.34 x LW (kg) + 19.7 x DMI (kg die^−1^) + 12

where: LW = Live Weight; DMI = Dry Matter Intake

Emission factors proposed by Husted (41), Amon et al. (42) and Kinsman et al. (43), which calculate, respectively 15.5 kg CH_4_ LU^−1^, 0.609 kg N_2_O LU^−1^, and 381 L CO_2_ LU^−1^ were used to assess the direct emissions from liquid and solid manure. Manure management is conducted through solid and liquid systems. Therefore, Tier 2 (44) was applied to estimate direct and indirect N_2_O emissions at field level and from leaching and runoff (45). The emission factors used for direct N_2_O were 0.02 (solid storage) and 0.001 (liquid storage) (45); indirect N_2_O emissions at field level were determined applying the emission factor of 0.01 N_2_O-N kg^−1^ of volatilized N (46), 0.0075 N_2_O-N kg^−1^ of N is lost through leaching and runoff (44) with a fraction of total N of 0.26 (47). Phosphorus loss in the form of phosphate (${\text{PO}}_{4}^{3-}$) was estimated as proposed by Nemecek and Kagi (48).

The quantity of P excreted in manure was estimated by the equation developed by Hollman et al. (49):

P_e_ = MY (kg d^−1^) × 0.781 + 50.4

where: MY, Milk Yield

NH_3_ emissions for excreted manure in the meadows were estimated at 5.7 g LU^−1^ day^−1^ (42), while the estimations for manure management were: 552 g ton^−1^ for slurry and 205 g ton^−1^ for solid manure in summer and 249 g ton^−1^ for slurry and 201 g ton^−1^ for solid manure in winter.

Regarding eutrophication effects, N leaching at field level was calculated by estimating the amount of N excreted by livestock: this quantity is function of the protein level of the ration, which is determined as the sum of urinary and fecal nitrogen.

Urinary N was estimated following the equation developed by Burgos et al. (50):

UN (g die^−1^ UBA^−1^) = −37.33 + 16.01 × MUN (mg dL^−1^)

where the average value of MUN (Milk Urea Nitrogen) considered for 1 kg of milk is 15.76 md dL^−1^ (50.51). With reference to fecal N, this is determined using the following equation by Jonker et al. (51) and Bianchi et al. (52):

Fecal N = Ingested N—(Urinary N + Milk N)

where: Ingested N is ~16% of crude protein and Milk N is 28% of Ingested N (52).

The water consumption (WC) of cows for each scenario was calculated with the equation presented in Meyer et al. (53):

WC (kg die^−1^) = −26.12 + (1.516 × AET) + [1.299 × milk kg (kg LU^−1^ die^−1^)] + (0.058 × LW) + (0.406 × Na (g LU^−1^ day^−1^)

where: AET, Average Environmental Temperature; LW, Live Weight; Na, quantity of sodium ingested daily by cows, estimated in 66.2 g day^−1^ (54).

#### Software and Impact Categories

The estimation of emissions occurring throughout the phases of the Toma di Lanzo cheese cycle production for the 4 scenarios, was carried out with the assistance of SimaPro 8.5 software and the Ecoinvent database (45, 55). The ReCiPe Midpoint method (European Hierarchist version 1.13) was used for the LCA analysis and the impact categories considered are shown in Table 2.

**Table 2:** Impact categories considered in LCA analysis.

**Impact category**	**Unit**
Climate change	kg CO_2_ eq
Freshwater eutrophication	kg P eq
Marine eutrophication	kg N eq
Natural land transformation	m^2^
Water depletion	m^3^
Fossil depletion	kg oil eq

### Economic Analysis

The goal of the economic analyses was to assess the costs and profitability of each scenario. For the cost analysis, the so-called conventional LCC scheme was adopted as it is well-suited for this purpose, as also acknowledged by other authors (56). This tool makes it possible to perform an in-depth analysis of the costs incurred throughout all phases of a product life cycle. In the case examined, all the costs of the rearing, milking, cheese-making and transport phases were analyzed with reference to 1 kg of Toma di Lanzo cheese for the four scenarios.

The data used were collected by directly interviewing the entrepreneurs and an average cost value for each input was used in the analysis. For all cost items, we refer to the purchase price of the inputs except for the labor factor. In this case, the family labor is in practice paid at opportunity cost.

**Figure 2:**
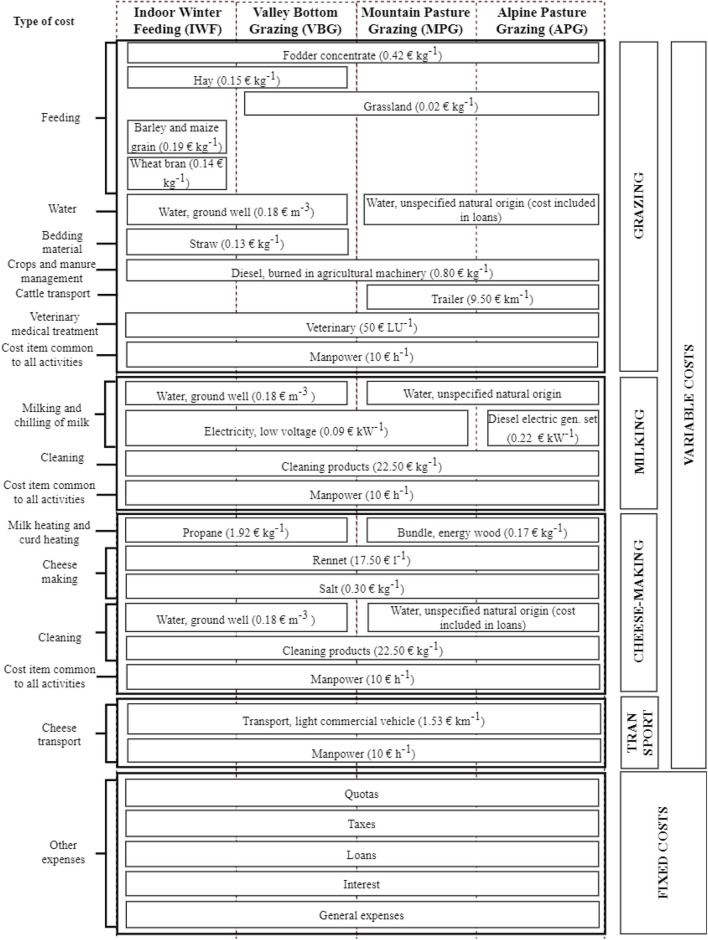
Cost items and price of inputs.

Figure 2 shows the main cost items divided by phase and referring to each scenario. In addition, costs have been calculated in relation to the type of cost (i.e., livestock feed, manure management, cleaning, etc.). The inputs necessary for the production of Toma di Lanzo cheese generate variable and fixed costs. Variable costs are partly common to the different scenarios and partly specific to each scenario.

Fixed costs refer to a set of items: quotas on capital invested in machinery and infrastructure, taxes, land and pasture rents, interest and overheads. With regard to interest, this item includes the assets owned by the entrepreneurs and employed in agricultural activities and have been remunerated at opportunity cost considering an interest rate of 1.0% for land use, 3.0% for capital goods, in agreement with Blanc et al. (57). The overheads item includes labor charges and contributions, the annual membership fee of the consortium for the protection of the Toma di Lanzo cheese and administrative costs.

For the profitability analysis, the sales price of Toma di Lanzo cheese in 2019 was considered as the reference price, equal to 9.00 € kg^−1^.

### Allocation Methods

In accordance with other authors (45, 58), an economic allocation method was chosen for the environmental and economic analyses. The allocation factor was considered to be the ratio between the economic value of the cheese sold and the total values of the outputs. The outputs considered are cheese (9.00 € kg^−1^), obtained from milk processing, calves (3.80 € kg^−1^) and cows (1.15 € kg^−1^). These values were multiplied by the volumes sold and an allocation factor of 0.87 for the cheese and 0.13 for the other outputs was obtained.

### Calculation of Feed Efficiency Indicators

The human-edible feed conversion efficiency for gross energy (GE) and crude protein (CP) was calculated as the ratio between the human-edible content in the produced milk (output) and the potential human-edible content of the consumed feedstuffs (input) (37). Net food production (MJ of GE/d and g of CP/d) was defined as human-edible output (MJ of GE and g of CP in the milk) minus human-edible input (38). The two indexes were calculated using the proportion of potential human edibles in feedstuffs proposed by Wilkinson (59). Data on GE content of feedstuffs were retrieved from the INRA (60) database. To calculate the energy content of the milk, the formula described by Herdt (61), including fat, protein, and lactose content of the milk, was used with the factor 4.184 for the conversion of calories to joules.

## Results

### Life Cycle Assessment Results

**Figure 3:**
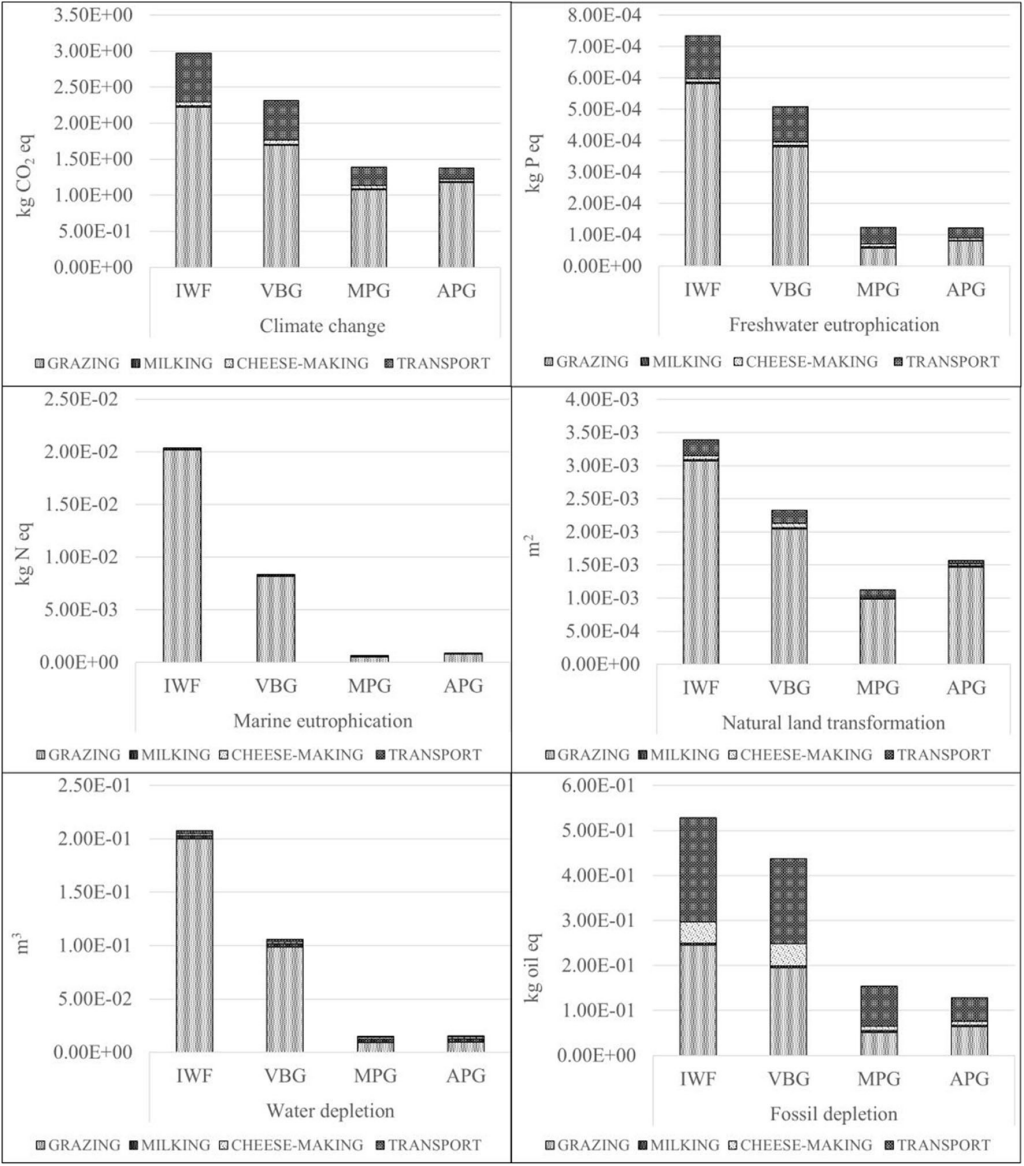
Environmental results.

Figure 3 shows the LCA results for each scenario, by making a distinction between the four life cycle phases and taking into account the six environmental impact categories considered in this study.

The IWF scenario reveals the highest level of impacts in all the considered categories. More specifically, it has the highest values in the grazing and transport phases. The VBG scenario impacts vary from 40 to 82% of IWF values. The mountain scenarios (APG and MPG), when compared to lowland scenarios (VBG and IWF), have a lower environmental impact: their incidence is never higher than 50% of IWF values. That is particularly evident for freshwater eutrophication, marine eutrophication and water depletion. Thus, it appears that, in those scenarios, there is a more sustainable use of the “water” resource. The environmental impact of APG and MPG scenarios is quite similar for all categories, except for natural land transformation (APG>MPG) and fossil depletion (APG < MPG).

The grazing phase causes the greatest environmental impact when compared to the other three phases. The incidence of this phase increases progressively from mountain scenarios (APG and MPG) to lowland scenarios (VBG and IWF) for all environmental impact categories. However, it is necessary to make some distinctions: APG is always higher than MPG, meanwhile IWF is always greater than VBG. Cattle nutrition plays an important role: essentially, in the APG scenario, farmers offer a higher amount of concentrate to lactating cows than in the MPG setting, due to the lower nutritional value of those grasslands and in connection to lactation. In the IWF scenario, feeding is based on dried fodder, cereal grains and concentrated feed whose use causes a higher environmental impact than grassland use.

Transport is the second most impacting phase in Toma di Lanzo production system for all impact categories. Moreover, VBG and IWF scenarios have a higher incidence than APG and MPG scenarios, due to the greater distance from the retail stores. The two environmental impact categories most affected by this phase are climate change and fossil depletion.

The cheese-making phase shows a relatively high environmental impact in fossil depletion. As in the previous phases, the incidence of environmental impact is higher in VBG and IWF scenarios than in APG and MPG settings. The difference between the scenarios mostly depends on how the cheese curd is heated. In APG and MPG settings, cheesemakers use firewood as heating energy, while in VBG and IWF they use propane. Thus, in the mountain settings, there is a greater use of natural and renewable resources than in the lowlands.

The milking phase ranks lowest in terms of environmental impact, with the exception of water depletion, due to a greater use of water in cleaning activities.

### Economic Results

Table 3 shows the profitability for the four scenarios examined, highlighting the costs for each phase.

**Table 3:** Economic results [€ kg^−1^].

		**Scenarios**			
**Phase**	**Type of cost**	**Indoor winter feeding (IWF)**	**Valley bottom grazing (VBG)**	**Mountain pasture grazing (MPG)**	**Alpine pasture grazing (APG)**
	**Variable costs**				
Grazing		**5.94**	**4.60**	**2.81**	**3.97**
	Feeding	3.49	1.45	1.07	2.10
	Bedding material	0.49	0.63	-	-
	Manure management	1.67	1.86	0.11	0.14
	Veterinary treatment	0.29	0.29	0.29	0.43
	Cattle transport to the mountain pasture	-	-	0.75	0.71
	Herd surveillance	-	0.38	0.58	0.59
Milking		**0.14**	**0.14**	**0.12**	**0.15**
	Milking	0.03	0.03	0.03	0.06
	Chilling milk	0.02	0.02	-	-
	Cleaning	0.09	0.09	0.09	0.09
Cheese-making		**0.48**	**0.48**	**0.43**	**0.43**
	Heating milk in boiler	0.12	0.12	0.08	0.08
	Cheese making	0.12	0.12	0.12	0.12
	Curd heating	0.07	0.07	0.06	0.06
	Cleaning	0.17	0.17	0.17	0.17
Transport		**0.10**	**0.10**	**0.07**	**0.08**
	**Fixed costs**				
Other expenses		**2.08**	**2.30**	**2.35**	**2.49**
	General expenses	0.32	0.32	0.32	0.32
	Quotas	0.96	0.96	0.96	0.96
	Taxes	0.05	0.05	0.05	0.05
	Loans	0.23	0.34	0.69	0.81
	Interests	0.52	0.62	0.33	0.34
Total costs		**8.75**	**7.63**	**5.77**	**7.11**
Revenues		**9.00**	**9.00**	**9.00**	**9.00**
Profit		**0.25**	**1.37**	**3.23**	**1.89**

*The bold values indicate the total costs for each phase*.

The mountain pasture scenarios (MPG and APG) allow better economic results to be achieved than the two valley bottom scenarios (IWF and VBG).

In the IWF and VBG scenarios, the costs with the greatest impact in the grazing phase are those related to feeding lactating cows and the management of livestock manure. In the MPG and APG scenarios, the costs for transporting the herd to the mountain pastures and for guarding the herd have a strong impact.

The differences in feeding costs can be explained by considering the different use of concentrate in the daily ration in the scenarios considered. This practice is necessary in the APG scenario to support nutritional requirements during lactation, which is in a declining phase in summer. On the other hand, the MPG scenario determines the lowest costs as the herds are present in two periods:

- in spring, when the grass is more nutritious and the pastures are more productive; the supplementation with concentrate is minimal but necessary to support high milk production;
- in autumn, when the cows are close to calving and are fed only grass and hay. The high costs of the IWF and VBG scenarios are linked to the fact that the enterprises need to source external inputs, incurring higher costs than in the case of self-production of forage or free grazing.

With reference to the milking phase, the APG scenario has high labor costs, as milking is performed manually. Conversely, the VBG and IWF scenarios have energy costs for milk cooling, which are not present in the mountain pasture scenarios, as cooling takes place by immersion of the tanks in spring water.

In the cheese-making phase, the higher costs in the VBG and IWF scenarios depend on the use of propane for curd heating, whereas in the other two scenarios a wood-fired boiler is used.

The costs of transporting the cheese to the point of sale depend on the average distance the cheesemaker has to travel, which is greater in the VBG and IWF scenarios than in the APG and MPG scenarios.

The fixed costs are higher in the two mountain pasture scenarios (APG and MPG) because the pasture rental has a strong impact on the farm costs. In contrast, in the two valley scenarios, the higher costs are determined by the interest on the capital invested by the entrepreneur in the business.

The comparison between the four scenarios examined shows that the MPG scenario determines the best economic results. In fact, the costs have a 64% incidence on revenues. This value increases in the APG scenario to 79% and in the VBG scenario to 85%. The IWF scenario, on the other hand, shows costs equal to 97% of revenues, highlighting the inefficiency of this production system.

From the overall examination of the results, it can be deduced that the APG scenario could improve its competitiveness, above all by reducing the use of human labor in livestock surveillance.

In the valley scenarios, the cost reduction could pass through an efficient management of livestock manure, maybe by adopting highly mechanized techniques, and through a reduction of energy costs in the cheese-making phase. Additionally, in this case it could be more profitable to switch from the use of non-renewable to renewable sources to heat milk and curd.

### Feed Efficiency Indicators

**Figure 4:**
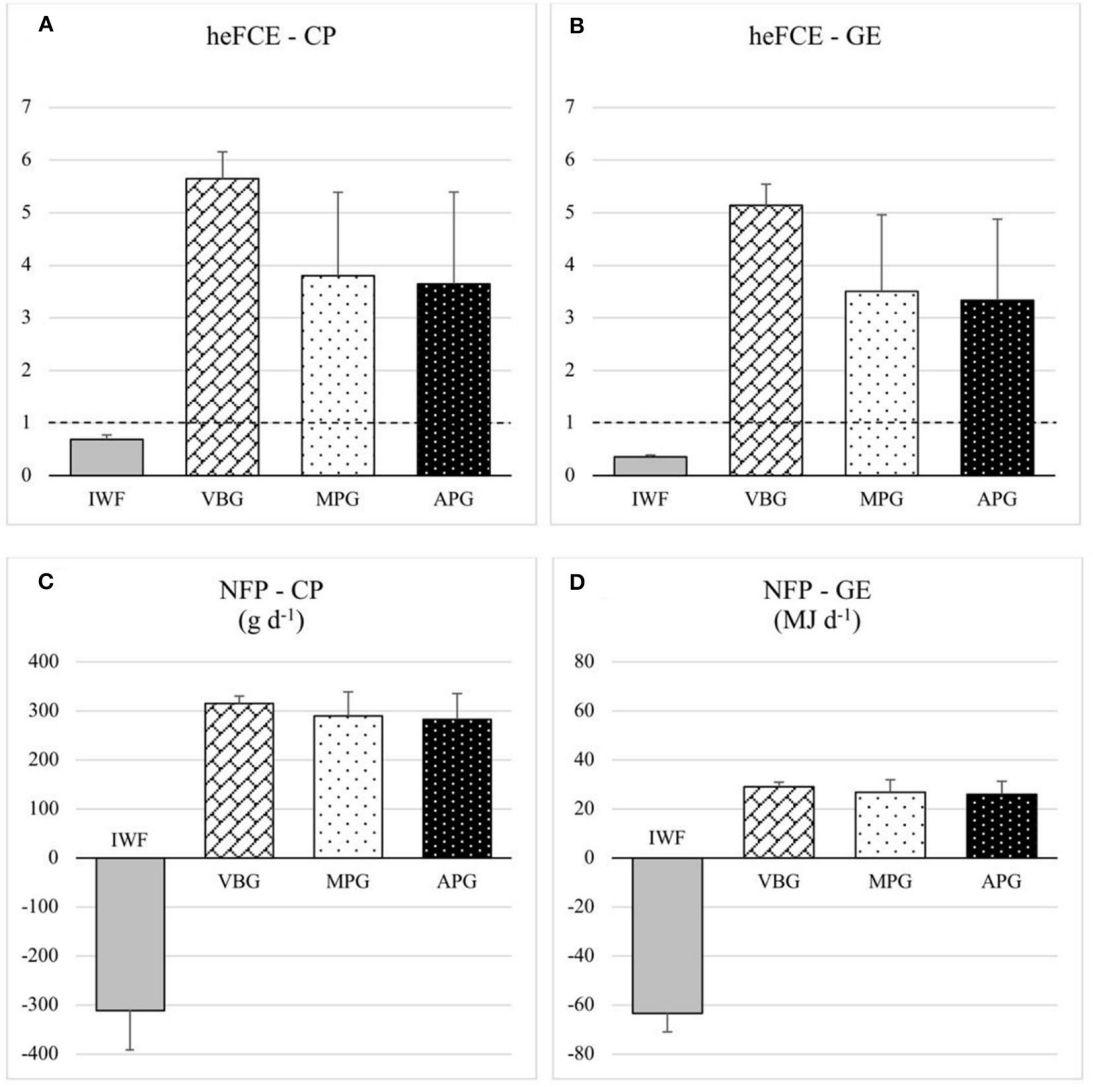
Human-edible feed conversion efficiency - heFCE **(A,B)** and net food production - NFP **(C,D)** for crude protein (CP) and gross energy (GE) comparing the four scenarios (IWF, Indoor Winter Feeding; VBG, Valley Bottom Grazing; MPG, Mountain Pasture Grazing; APG, Alpine Pasture Grazing); the dashed line in **(A)** and **(B)** set at 1.0 indicates the efficiency threshold.

The calculated human-edible feed conversion efficiency (heFCE) for the four scenarios are shown in Figures 4A,B for protein and energy, respectively. The dotted horizontal line in the graphs indicates a heFCE of 1. For farms that did not offer additional feed supplement to grazing cows, human-edible inputs were zero, thus the heFCE could not be calculated (division by zero). Average heFCE for protein ranged from 0.69 for IWF up to 5.65 for VBG, with MPG and APG values equal to 3.80 and 3.65 points, respectively. Similarly, IWF showed the lowest average heFCE for energy (0.36), followed by MPG and APG (3.51 and 3.33, respectively), while VBG had the highest index (5.14). All the calculated heFCE indexes, both for energy and protein in VBG, MPG and APG scenarios, were largely higher than 1. Conversely, IWF had an heFCE value below 1, both for protein and energy.

The results of net food production (NFP) for the four scenarios are shown in Figures 4C,D for protein and energy, respectively. The differences between the four scenarios had also strong effect on the NFP. The calculations for the considered scenarios revealed positive NFP in VBG, MPG, and APG scenarios, both in terms of protein (315.08, 289.68, and 282.67 g d^−1^, respectively) and energy (29.08, 26.84, and 25.99 MJ d^−1^, respectively). Conversely, the NFP for IWF was negative, both for protein and energy (−311.19 g d^−1^ and −63.36 MJ d^−1^, respectively).

## Discussion

### Environmental Implications

The environmental impact assessment shows that the two mountain scenarios (APG and MPG) for the Toma di Lanzo cheese production achieve more sustainable environmental performances when compared to the lowland contexts examined (IWF and VBG).

In all scenarios, the greatest impact is determined by the grazing phase for all environmental impact categories, with the exclusion of fossil depletion. Climate change is one of the most evaluated environmental impact indicators both in the agricultural and dairy sector (62–64), and the grazing phase determines the largest contribution in the four scenarios, especially in the VBG and IWF scenarios.

However, these values are below the average emissions recorded in other works (65, 66), given the high self-sufficiency and the low dependence on purchased feed in the Toma di Lanzo production process. Our results could also be justified by considering previous research of Guerci et al. (23). In that study, a comparison between the different dairy farms showed that a high efficiency of food conversion by animals was more effective in terms of environmental impact and, above all, in the production of greenhouse gases and the use of non-renewable energy. Therefore, aspects such as animal welfare, for example in extensive livestock management (more space available, access to pasture, quantity and quality of fresh forage, air quality) (67, 68), could indirectly affect the environmental impact by operating on animal efficiency. At the same time, fresh forage may lead to a greater food conversion index and food efficiency (69), providing a positive impact on greenhouse gas emissions and on the environmental influence of livestock farming (70). Therefore, the environmental efficiency of the mountain systems studied is provided by a good quality of the pasture, as also argued by other authors (71, 72), and by an excellent efficiency in the use of the feed by the animal which, incidentally, is influenced by an intrinsic vocation of the breed.

The results of the LCA define a higher environmental sustainability profile of both low-input scenarios, especially when considering water pollution and use (freshwater eutrophication, marine eutrophication and water depletion), in comparison to the high-input scenarios. Relating to water use and pollution, grazing appears to be the most impactful phase, with the exception of water depletion, for which the milking phase is the most unsustainable. Canellada et al. (73) found similar results in their study, evidencing that milk production in small-scale cheese factories affects marine eutrophication, freshwater and marine ecotoxicity. Furthermore, Nemecek and Alig (71) described the environmental efficacy of low-input mountain production scenarios, producing results comparable to ours in terms of acidification, eutrophication potentials and resource consumption.

Likewise, Penati et al. (74), demonstrated how farms with a low stocking rate with high feed self-sufficiency, similar to the mountain scenarios we studied, showed good performances in terms of acidification and eutrophication, supporting the results of our research. In addition to the environmental significance related to water use, APG and MPG contribute to the maintenance of water resources by reducing competitiveness with humans.

Natural land transformation is one of the most impactful categories for the two mountain systems, confirmed by another research in which the environmental impacts, deriving from low-input cheese making systems, mainly originated from natural land transformation (73). However, in the studied context, land use by animals and the transformation of pastures take on important positive meanings in the Alpine context, with positive effects on biodiversity, landscape quality and soil conservation (74, 75). These considerations enable to affirm how the survival of production systems such as Toma di Lanzo cheese is strictly dependent on the territory and the animal breeds associated with it.

Therefore, Toma di Lanzo represents an example of a sustainable production system, where our results could be further improved by orienting production toward ecosystems and the use of mountain resources. In this case, the use of local, low-yielding breeds, which maximize the resource efficiency of the territory, belies the theories in which the use of pastoral dairy production systems on grassy pastures reduces emissions by manipulating the diet of livestock or controlling emissions from agricultural waste treatment plants (76). In the production of Toma di Lanzo the unique relationship between animals and the environment improves production sustainability. The use of hardy breeds makes it possible to maximize the feed conversion index and exploit the grazing resources. The existence and survival of farming systems for mountain livestock depends on these breeds, which, indiscriminately, have a positive impact on sustainability aspects such as the conservation of biodiversity.

### Economic Implications

The economic analysis shows significant differences between the four considered scenarios both in terms of costs and profits in the Toma di Lanzo cheese life cycle. Our results confirm—as stated by other authors (77–79)—that feeding is the most significant cost item among the variable costs. That is evident in the IWF and VBG scenarios, confirming, on the contrary, how the exploitation of pasture resources contributes to a concrete reduction of farm costs. In fact, in the high-input livestock systems, feeding costs normally account for ~60% of total costs, as has been evidenced in other geographical settings (80, 81). In the two low-input scenarios, this item represents a lower cost in relation to the overall production costs (82). This result is in line with other literature, that highlight the association between pasture use and costs of production, underlining a linear profit decline when including externally purchased feeds (82, 83).

Another aspect to be taken into consideration, is the heavy dependence of intensive livestock farming on purchased feedstuffs, which closely interrelate farm costs and market trends (79, 83). On the contrary, the Toma di Lanzo cheese production model, in the MPG and APG scenarios, manages to overcome the raw material price fluctuations, which affect the economic efficiency of high input systems (such as the IWF and VPG scenarios) (78, 82, 84). Therefore, scenarios like APG and MPG are mostly self-sufficient, with the possibility to maintain sustainable production costs and higher long-term economic efficiency.

Therefore, we highlight the importance of the development of breeding systems with a low level of extra farm input, offering the prospect of developing other productions systems, similar to the one we studied. Moreover, the Toma di Lanzo cheese—strongly dependent on the resources provided by the Alpine ecosystem—has a positive impact on the survival of these areas, as well as the maintenance of production traditions, evidently providing relevant eco-system services (81). This symbiotic relationship between product-animal-environment, in addition to the positive impact on environmental and social sustainability, proves to play an important role in economic aspects for farm survival (82, 85).

In general, mountain livestock systems have several critical issues mainly linked to social factors such as low generational turnover, workloads, modest life quality of families (86–88). A desirable improvement of the farmers' quality of life and concurrently of the competitiveness of these enterprises, in our opinion, can be implemented through better manpower management. A possible solution could be the reduction in family-based farming activities (by replacement with salaried workers) and, at the same time, an increase in off-farm activities for household members (87, 89), with the transition to a form of direct sale of the cheese, at farmers markets or through solidarity-based purchasing groups. This business direction could allow a higher remuneration of the product to be obtained, driving a greater appreciation of this cheese obtained from grazing in mountain and Alpine environments. In fact, Toma di Lanzo cheese is currently sold at points of sale at an agreed price, while the changeover to direct sale would make it possible to obtain a premium price linked to the mountain production added value (as occurs for other typical Italian mountain products, PDO, PAT) (90).

### Feed Efficiency Implications

Livestock is considered a major contributor to global environmental issues (91). However, the livestock sector has already achieved some significant improvements in reducing its environmental impacts over the last decades (92, 93). Sustainability is a complex concept that includes various dimensions, which in turn involve several aspects. One of the sustainability themes that has recently seen an increasing interest is the food-feed competition (i.e., the use of potential human food in livestock feeding), especially in ruminant nutrition (94). The feed efficiency indicators (heFCE and NFP) allow the assessment of the potential human-edible content in animal diets, providing an index of sustainability.

The results obtained in the present study are consistent with previous studies about increased efficiency (from a human-edible production point of view) when dairy cows are fed forage and by-products. Ertl et al. (37) first showed a positive correlation between heFCE and the grassland area in Austrian dairy farms. Laisse et al. (95) estimated that cows fed grass and small amounts of concentrate can produce 2.5 times more human-edible protein than they consume. Comparing different dairy systems, Dentler et al. (96) detected significantly higher heFCE values for low-input grass-based farms compared to high-input confinement-based farms, both in terms of crude protein (3.30 vs. 0.76, respectively) and energy (2.95 vs. 0.69, respectively).

Although cows in IWF settings consumed relevant amounts of conserved forages, representing significant human-inedible portion of the diets, that scenario showed the lowest indexes when compared to the other scenarios. As suggested by previous studies (38, 97), not only the amount of concentrate in the diet, but also its composition strongly affects heFCE and NFP. The inclusion of by-products constitutes an effective way to reduce the human-edible portion in cow diets during the wintertime, when the climatic conditions in mountain regions do not allow grazing activity.

In conclusion, human-edible indicators confirm that grazing and grass-based feeding systems are one of the most sustainable ways to produce milk (98). Replacing cereal grains and pulses in the diet with by-products reduces food-feed competition and thus further improves the sustainability of traditional dairy systems in Alpine regions.

## Data Availability Statement

The raw data supporting the conclusions of this article will be made available by the authors, without undue reservation.

## Author Contributions

TV, SB, VM, and PC wrote the manuscript. LMB critically reviewed and commented on the manuscript. All authors contributed to manuscript revision, then read, approved the submitted version, and contributed equally to conceptualization.

## Conflict of Interest

The authors declare that the research was conducted in the absence of any commercial or financial relationships that could be construed as a potential conflict of interest.
